# Toluene Inhalant Addiction and Cardiac Functions in Young Adults: A Comparison of Electrocardiographic and Echocardiographic Parameters

**DOI:** 10.1002/clc.70037

**Published:** 2024-10-24

**Authors:** Gürkan Karaca, Erdinç Hatipsoylu, Ahmet Ekmekci, Ömer Kamil Yazıcı, Ali Kimiaei, Seyedehtina Safaei, Ahmet Bilge Sözen

**Affiliations:** ^1^ Department of Cardiology Bahçeşehir University Faculty of Medicine Istanbul Turkey; ^2^ Department of Cardiology VM Medical Park Maltepe Hospital Istanbul Turkey; ^3^ Department of Cardiology Dr. Siyami Ersek Thoracic and Cardiovascular Surgery Educational Research Hospital Istanbul Turkey; ^4^ Department of Cardiology Medical Park Pendik Hospital Istanbul Turkey; ^5^ Department of Medical Oncology Ordu University Training and Research Hospital Ordu Turkey; ^6^ Department of Internal Medicine Istanbul University, Faculty of Medicine Istanbul Turkey

**Keywords:** arrhythmia, echocardiography, electrocardiography, P wave, QT interval, thinner, thinner addiction, toluene

## Abstract

**Background:**

Volatile substance (thinner) addiction can cause serious cardiac events, such as malignant ventricular arrhythmias, acute coronary syndromes, sudden death syndrome, and dilated cardiomyopathy, as reported in many case studies. We aimed to find echocardiographic and electrocardiographic parameters that could foresee these adverse outcomes in clinical settings.

**Methods:**

We enrolled 32 healthy young adult patients with at least 1 year of thinner addiction and no cardiac symptoms. We also recruited a control group of 30 healthy individuals without any medical problems. Both groups received standard echocardiography and ECG tests. We analyzed the following echocardiographic parameters: LVEDd (left ventricular end‐diastolic diameter), LVESd (left ventricular end‐systolic diameter), mitral valve EF slope, E/A ratio, and aortic and pulmonary valve VTI (velocity time integral). We also measured the corrected (QTc), uncorrected QT intervals, and widest P‐wave values in the ECG. We used the SPSS 13 software for statistical analysis.

**Results:**

The echocardiographic findings did not differ significantly between the groups. However, the ECG results showed that the thinner addicts had higher values of corrected (QTc), uncorrected QT intervals, and widest P‐wave values than the control group, according to Mann–Whitney *U* and Student's *T* test.

**Conclusion:**

Corrected QT (QTc) and P‐wave duration are increased in individuals with a thinner addiction. These findings may suggest a higher risk of sudden cardiac death, atrial, and ventricular dysrhythmias in the future.

## 1 Introduction

Volatile substances (inhalants) are chemicals that evaporate easily at room temperature [1]. They can be classified into three groups based on how they affect the body. The first group consists of volatile solvents; the second group includes gasoline and anesthetics; and the third group comprises alkyl nitrites that produce nitric oxide when vaporized. People use these substances for their quick and pleasant effects, such as mild euphoria and relaxation [2]. These substances are also cheap and widely available, making them prone to abuse, especially by children and teenagers.

Organic solvents are one type of volatile substance that is commonly used in various products, such as paints, polishes, adhesives, toner cartridges, correction fluids, and dry cleaning solutions [3, 4]. These products expose people to organic solvents through different routes, such as inhalation, skin contact, and ingestion [4]. After cigarettes and marijuana, volatile substances are the most frequently abused substances [1]. Most volatile substances have a depressant effect on the brain, similar to alcohol, except for nitrites [5].

People who intentionally inhale organic solvents are usually male and under 20 years old. There have been rare cases of solvent abuse among very young or very old people [6]. The common methods of inhalation are pouring the solvents into a plastic bag, inhaling from a bottle, or applying them to clothing or other surfaces.

The short‐term effects of inhalation are enjoyable and include euphoria, alertness, and disinhibition, followed by relaxation, dizziness, visual and auditory hallucinations, fatigue, and sleepiness [7, 8]. However, inhalation can also cause unpleasant symptoms, such as coughing, sneezing, increased salivation, skin reddening, nausea, vomiting, photophobia, disorientation, double vision, impaired coordination, speech difficulties, reduced reflexes, and nystagmus [8]. The long‐term effects of inhalation are harmful, affecting various organs and systems, such as the central nervous system, the blood system (methemoglobinemia, aplastic anemia, and leukemia), the heart (myocardial edema, irreversible myocarditis, fibrosis, and congestive heart failure), the liver, the upper respiratory tract, the lungs, and the kidneys [3, 8, 9]. Brain imaging studies have shown widespread brain damage, such as atrophy, ventricular dilation, and sulcus expansion, in chronic users [5].

The most serious risk of inhalation is sudden death, which can occur in acute poisoning or initial use. Sudden death can be caused by vagal stimulation, anoxia, respiratory depression, and cardiac arrhythmias. However, the most common cause is ventricular arrhythmias leading to cardiac arrest. Other causes, such as aspiration or trauma, can also be fatal [3, 8].

Thinner misuse is a growing problem that has health and social implications. Therefore, it is important to assess the health effects of thinner misuse and to raise awareness among the users, their families, and society. The cardiac effects of thinner are often documented in case reports in the literature. The aim of this study was to examine cardiac functions in young adults who were addicted to volatile substances (toluene) and did not have any symptoms and to detect any early signs of damage.

## 2 Methods

### 2.1 Study Group

The study recruited young adult patients with volatile substance addiction who were treated at the Department of Psychiatry of the Istanbul Faculty of Medicine. They underwent cardiac imaging (echocardiography and electrocardiography) at the Department of Cardiology of the same institution. The control group consisted of volunteers who matched the study criteria and agreed to have the same imaging tests at the Dr. Siyami Ersek Hospital, Department of Cardiology. The study had 62 participants (32 patients and 30 controls).

### 2.2 Inclusion Criteria for the Study

- At least 1 year of confirmed volatile substance use.
- No history of systemic diseases (such as diabetes, anemia, hypertension, chronic renal insufficiency, chronic lung disease, chronic liver disease, etc.).
- No evidence of cardiomyopathy, heart valve disease, arrhythmia, or previous cardiac surgery.

### 2.3 Exclusion Criteria for the Study

- Individuals who were not addicted to volatile substances.
- History of systemic diseases (such as diabetes, anemia, hypertension, chronic renal insufficiency, chronic lung disease, chronic liver disease, etc.).
- Presence of cardiomyopathy, heart valve disease, arrhythmia, or prior cardiac surgery.

The control group was composed of 30 young adults who had no history of volatile substance use, systemic illnesses, cardiomyopathy, heart valve problems, arrhythmias, or cardiac surgeries.

### 2.4 Echocardiographic Assessment

The study participants had echocardiography according to the American Society of Echocardiography guidelines, using a Vivid 7 echocardiography device (GE Vingmed, Horten, Norway) with a 2.5 MHz transducer in the left lateral decubitus position. The standard parasternal and apical windows were used to measure the size, thickness, and motion of the heart chambers and walls. M‐Mode measurements included the dimensions, thickness, and motion amplitudes of the heart chambers and walls, as well as the motion amplitudes of the mitral and aortic valves. Mitral EF slope was measured using a parasternal long‐axis view with 2D echocardiography. Doppler measurements included the flow velocities across the mitral, aortic, tricuspid, and pulmonary valves, as well as the systolic and diastolic time intervals.

### 2.5 Electrocardiographic Assessment

The ECGs of both the patient and control groups were recorded. QT parameters were measured after enlarging the recordings three times with a photocopy machine using a 12‐channel ECG device (Hewlett‐Packard M 1709‐A) at a paper speed of 50 mm/s (gain 10 mm/mV). The QT interval was measured from the start of the QRS complex to the end of the T wave. If the T wave was inverted, the point where the trace returned to the T‐P baseline was used, and if there was a U wave, the lowest point between T and U was used as the end of the T wave. If the end of the T wave was unclear, another recording was used for analysis. At least nine leads were analyzed for each patient. The minimum (QTmin) and maximum (QTmax) QT intervals were calculated. Each QT interval was corrected using Bazett's formula based on heart rate, with upper limits of 420 ms for males and 430 ms for females. The corrected QT interval (QTc) was calculated using the formula QT/√RR, with both QT and RR measured in seconds.

### 2.6 Statistical Analysis

Statistical analysis was conducted using the SPSS 13.0 software (Chicago, IL, USA). Continuous variables were presented as mean, standard deviation, median, and range. After confirming the assumption of normal distribution for continuous variables, independent sample *t* tests were employed to compare the distribution of continuous variables between groups, and Mann–Whitney *U* tests were used as a non‐parametric test for continuous variables that did not meet the assumption of normal distribution. In group comparisons, a *p* value less than 0.05 was considered statistically significant.

## 3 Results

Thirty‐two young adult patients who met the inclusion criteria were evaluated through echocardiography and electrocardiography, and they were compared with a control group consisting of 30 young adult individuals. No significant differences were observed between the groups in terms of gender, age, and heart rate (Mann–Whitney *U*; Table 1).

**Table 1:** Demographic characteristics.

Variable	Groups	*N*	Mean	Standard deviation	Male	Female	Total	*p* value
Age	Control	30	21.10	4.58	—	—	—	*p* > 0.05
	Patient	32	20.38	4.50	—	—	—	
Sex	Control	—	—	—	28 (93.3%)	2 (6.7%)	30	*p* > 0.05
	Patient	—	—	—	30 (93.8%)	2 (6.3%)	32	

The longest QT interval obtained from ECG records was calculated, and a statistical analysis between groups was performed without correction for speed (*T* test, *p* = 0.01), and the calculated *p* value was found to be significant (Table 2).

**Table 2:** Comparison of longest QT intervals between control and patient groups.

Variable	Groups	Mean	Standard deviation	*p* value
Longest QT (ms)	Control	360.00	30.91	*p* = 0.01
	Patient	388.44	29.19	

No difference in heart rates was observed between the patient and control groups (Student's *T* test, *p* > 0.05, Table 3).

**Table 3:** Comparison of heart rates between control and patient groups.

Variable	Groups	Mean	Standard deviation	*p* value
Heart rate (beats per minute)	Control	70.03	14.98	*p* > 0.05
	Patient	72.56	11.86	

Corrected QT (QTc) values according to the Bazett formula were found to be statistically significant (Mann–Whitney *U*, *p* = 0.003) (Table 4). The widest P values were compared, and it was observed that they had significantly increased in the patient group (Mann–Whitney *U*, *p* < 0.001) (Table 4).

**Table 4:** Comparison of corrected QT (QTc) and widest P values between control and patient groups.

Variable	Groups	Mean	Standard deviation	*p* value
QTc (ms)	Control	387.69	49.39	*p* = 0.003
	Patient	425.42	43.21	
Widest P (ms)	Control	108.67	9.90	*p* < 0.001
	Patient	118.28	10.89	

The echocardiographic measurements of LVEDd, LVESd, MV EF Slope, mitral valve E/A ratio, aortic valve VTI, and pulmonary valve VTI were conducted for both patient and control groups. No significant differences were observed between the groups (LVEDd; Mann–Whitney *U*, *p* > 0.05. LVESd; *T* test, *p* > 0.05. Mitral valve EF slope; *T* test, *p* > 0.05. Mitral valve E/A ratios; Mann–Whitney *U*, *p* > 0.05. Aortic valve VTI; *T* test, *p* > 0.05; pulmonary valve VTI, Mann–Whitney *U*, *p* > 0.05) (Table 5).

**Table 5:** Echocardiographic measurements comparison between control and patient groups.

Variable	Groups	Mean	Standard deviation	*p* value and test
LVEDd (cm)	Control	4.60	0.37	*p* > 0.05 Mann–Whitney *U*
	Patient	4.68	0.43	
LVESd (cm)	Control	2.95	0.34	*p* > 0.05 *T* test
	Patient	3.00	0.37	
Mitral valve EF slope (cm/sn2)	Control	12.37	2.59	*p* > 0.05 *T* test
	Patient	12.96	2.87	
Mitral valve E/A ratio	Control	1.76	0.41	*p* > 0.05 Mann–Whitney *U*
	Patient	1.67	0.49	
Aortic valve VTI (cm)	Control	22.42	4.87	*p* > 0.05 *T* test
	Patient	20.40	4.07	
Pulmonary valve VTI (cm)	Control	17.53	3.33	*p* > 0.05 Mann–Whitney *U*
	Patient	17.46	3.34	

Comparisons between the patient and control groups in terms of echocardiographic parameters did not reveal any significant differences (Table 5). Systolic and diastolic functions of the left ventricle were found to be well preserved in young adult individuals, irrespective of their substance dependence. Noteworthy findings, however, were observed in the ECG records of the patients. The uncorrected longest QT values were significantly prolonged in the substance‐dependent group (mean: 388.44 ms, standard deviation: 29.19, *T* test, *p* = 0.01, Table 3). Furthermore, the corrected QT (QTc) values, adjusted according to the Bazett formula, were also statistically significantly prolonged in the patient group (mean: 425.42, standard deviation: 43.21, Mann–Whitney *U*, *p* = 0.003) (Table 5). Additionally, the widest P‐wave values were significantly broader in the patient group, exceeding 110 ms (mean: 118.28, standard deviation: 10.89, Mann–Whitney *U*, *p* < 0.001, Table 5).

## 4 Discussion

In the literature, the clinical outcomes of acute, chronic exposure, or addiction to volatile substances (toluene) can be categorized under the following main headings:

### 4.1 Volatile Substances and Sudden Death

The most frequently emphasized cardiac adverse effect of volatile substances is their ability to cause deadly cardiac arrhythmias. Sudden deaths related to volatile substances can result from both the direct and indirect effects of these substances on the heart.

A study in the United Kingdom reported 140 deaths related to volatile substance abuse from 1971 to 1981. Of these deaths, 59% were directly caused by the toxic effects of volatile substances on the heart, while 41% were indirectly caused by volatile substance use (8% from trauma, 19% from suffocation due to plastic bags, and 14% from inhalation of stomach contents). This study highlights that the risk of sudden death from the toxic effects of volatile substances on the heart was higher for aerosols and lower for adhesives and solvents [10]. Furthermore, another study in the United States found an epidemic of sudden deaths involving 110 cases related to volatile substance use between 1960 and 1970. Autopsies of these cases did not show any anatomical defects that could explain the sudden deaths. Therefore, it was suggested that these deaths were related to ventricular arrhythmias [11]. The significant increase in the QTc value in the patient group in our study, without any structural heart disease, supports this suggestion.

Moreover, a study by Adgey and colleagues in the United Kingdom shows the rising incidence of deaths related to volatile substances. According to their research, most of the victims of volatile substances in the United Kingdom are males, with the highest rate in the 15–19 age group. The researchers found that butane, propane, lamp oil from fuel gases, correction fluid and dry cleaning solutions from solvents, and fire extinguishers accounted for 60% of these deaths. The authors propose that volatile substances make the heart more sensitive to catecholamines, which can trigger sudden death when there is sudden stimulation or exertion. The main mechanism in this proposal is cardiac arrest, but other factors that can contribute to sudden death are respiratory depression, anoxia due to vomiting induced by vagal stimulation, aspiration, or injuries. The researchers also reported that butane may cause laryngeal edema or spasm. They recommend avoiding immediate intubation when there is a cardiac‐respiratory arrest after exposure to butane. Moreover, the study stresses the importance of a conservative approach to volatile substance‐related ventricular tachyarrhythmias, the use of beta‐blocker agents to protect the catecholamine‐sensitive hearts in the early stages of resuscitation, and the avoidance of sympathomimetic agents [12].

Additionally, Obara et al. described three cases of acute toluene intoxication that occurred in Ube City, Japan, between 1996 and 1997. All three patients were male, and one of them died 2 days after hospitalization. The patient who died had impaired consciousness, tachycardia, low blood pressure, and dyspnea when he arrived at the hospital. He also had lung crackles, high liver enzymes, severe muscle weakness, absence of deep tendon reflexes, stupor, atelectasis on chest X‐ray, bilateral pleural effusion, decreased voltage in all ECG leads, and signs of myocardial damage. His blood and urine toluene levels were much higher than those of the other two patients [13].

People who abuse volatile substances often use paint thinner or adhesive. Paint thinner has aromatic hydrocarbons such as xylene, toluene, and N‐hexane. Adhesive has toluene, benzene, ethyl acetate, and methylene chloride. Halifeoğlu et al. showed that paint thinner is broken down by cytochrome P‐450 enzymes in the body, which produce free radicals. They also found that people who make paint thinner have more malondialdehyde, a marker of lipid peroxidation, in their blood and more antioxidant enzymes like glutathione peroxidase and superoxide dismutase in their red blood cells. This means that paint thinner causes oxidative stress and activates antioxidant defenses [14].

There are reports of toluene ingestion causing hypokalemia, respiratory suppression, metabolic acidosis, and ventricular tachycardia. These symptoms improved after dialysis [15]. Another report showed that a person who inhaled toluene (adhesive) had severe sinus bradycardia, which went back to normal after the toluene wore off [16]. This case shows the relevance of the QTc prolongation that we found in our study.

### 4.2 Volatile Substances and Myocardial Infarction

The SHEEP study [17] was one of the most important studies that looked at how volatile substances affect the heart. It included 2993 people who were 45–70 years old. It showed that exposure to dynamite explosions or organic solvents increased the risk of myocardial infarction by 1.5–2 times, depending on the duration and intensity of the exposure. Some cases in the literature agree with this study [18, 19].

Volatile substances probably induce myocardial infarction due to vasospasms, invariably accompanied by ventricular fibrillation (VF) and, in some instances, persistent VF attacks. Vural et al. [20] reported a case of a patient exposed to a high concentration of paint thinner in a confined space who experienced severe chest pain just 15 min after exposure. An ECG at the emergency room showed acute inferior myocardial infarction. This case also featured resistance to defibrillation, and despite antiarrhythmic treatment, recurrent VF episodes were observed. The authors believe that in addition to ischemia resulting from toluene‐induced vasospasm, the direct cardiotoxic effects of paint thinner may have contributed to the formation of this life‐threatening arrhythmia. In this case, recurrent VF episodes occurred within the first hour, and no signs of ischemia were found in the initial 8 h. Subsequent stress ECG tests did not indicate ischemia, and during a 2‐year follow‐up, there were no symptoms or findings suggestive of ischemia.

Lately, there have been reports of acute myocardial infarctions in other cases involving the inhalation of toluene vapors. Notably, in one individual who abused toluene vapor, the development of recurrent myocardial infarctions without Q‐waves is intriguing [21, 22].

### 4.3 Volatile Substances and Heart Failure

There is not much literature about how volatile substances affect heart failure. We will talk about a few cases here.

In a 14‐year‐old male patient who had sniffed chronic trichloroethylene (dry cleaning fluid), atrial flutter was observed in conjunction with congestive cardiomyopathy. After the cessation of exposure, his condition improved. The author suggested that trichloroethylene might enhance the effects of catecholamines and postulated that cardiomyopathy could have an ischemic origin [23]. In another report, the first patient, who sniffed 1,1,1‐trichloroethane before, had VF during surgery when given halothane, which is similar to what he was exposed to. He had mild heart failure after that. The second case, a patient with mild, stable heart failure from being exposed to 1,1,1‐trichloroethane at work, had end‐stage heart failure when given halothane for hernia surgery. The author pointed out the possible toxic effect of halothane on people who were exposed to 1,1,1‐trichloroethane before [13].

Several researchers have previously reported that thinner, due to coronary vasospasm, can lead to apparent ischemia, resulting in myocardial infarction and/or ventricular fibrillation [18, 20, 21, 22]. In these patients, the resistance to defibrillation and the occurrence of recurrent ventricular fibrillation attacks, despite antiarrhythmic treatment, are noteworthy. Beyond ischemia resulting from vasospasm, it is suggested that toluene's direct cardiotoxic effects also play a role in the development of arrhythmias. In the case of thinner‐induced cardiomyopathy, it is believed that thinner contributed to the development of cardiomyopathy by causing silent ischemia through vasospasm [24]. The matter of why some cases of volatile substance abuse lead to evident ischemia while others silently progress to cardiomyopathy remains unknown. However, the amount of the substance, how long they are exposed, and some genetic and environmental factors that enhance the cardiotoxic effects of the substance may play a role in these different responses.

A second case of cardiomyopathy resulting from toluene exposure has been reported from Poland. In this case, atrial fibrillation and congestive heart failure were diagnosed. The ejection fraction was initially at 15%, but it increased to 45% after treatment and cessation of exposure [25]. Vural et al. presented a case of dilated cardiomyopathy in a 21‐year‐old male patient who had been intermittently using toluene for 10 years. The patient complained of exertional dyspnea, easy fatigue, abdominal distension, and an irregular pulse. Tests showed an incomplete right bundle branch block on the ECG, hepatomegaly, left ventricular dilatation, and global hypokinesis with an ejection fraction of 40%. Follow‐up at 6 months after heart failure treatment indicated an improvement in pathological findings [24].

The QT interval serves as a measure of cardiac depolarization and repolarization [26]. QT and QTc intervals are also significant determinants of clinical morbidity and mortality [27]. Therefore, a comprehensive examination of factors influencing the QT interval is of great importance. Numerous factors can influence QT and QTc intervals, as well as QT and QTc dispersion. Several anthropometric and autonomic factors affecting the QT interval have been identified, both in childhood and adulthood. It has been revealed that these factors can lead to notable changes and differences in the QT interval, even in the absence of metabolic diseases or electrolyte imbalances. Many studies have reported conflicting results [28, 29]. Age is a prominent factor under scrutiny. While some studies have demonstrated a lengthening of the QT interval and an increase in QTc dispersion with age [28], others have indicated that age may not have a significant impact [30, 31]. Studies have shown an inverse relationship between age and QTc in children [32], and in the adult population, gender has been identified as the significant factor in the relationship between age and the QT interval [29, 32]. All studies related to gender have consistently demonstrated that the QT interval is longer in females, and consequently, women are at a higher risk than men in clinical conditions such as long QT syndrome triggered by drugs [33, 34]. The contribution of hormonal factors to gender differences has also been explored [35, 36]. In our study, both the patient and control groups comprised young adult individuals. The QTc interval was within normal limits in the control group. Due to the limited number of female participants, an evaluation based on gender was not performed.

Studies investigating QT and QTc values in thinner abusers are relatively scarce in the literature. Alper et al. assessed QT intervals in 44 individuals who abused toluene through inhalation, comparing those who experienced syncope with those who did not. Their findings revealed that, compared to the control group, the QT interval and corrected QT dispersion were increased in both syncope and non‐syncope toluene users. Notably, these intervals were even more prolonged in those who experienced syncope [37]. It is essential to note that these individuals had a history of unexplained syncope, irrespective of neurological or cardiac causes. In our patient group, no symptoms were reported, yet an elongated QTc interval was observed, consistent with the findings of Alper et al., where no echocardiographic pathology was detected in both the patient and control groups.

In our study, the group of thinner users who did not have any heart problems or birth defects had longer QTc values (mean: 425.42, standard deviation: 43.21, Mann–Whitney *U*, *p* = 0.003). This supports the case reports and clinical descriptions in the literature. It is essential to consider that QTc prolongation in asymptomatic, thinner addicts could lead to ventricular arrhythmias. Many case reports and experiments in the literature [15, 16, 37, 38, 39] have indicated that acute or chronic exposure to thinner may lead to ventricular arrhythmias. However, there have been no predictive studies regarding these adverse outcomes in asymptomatic, thinner addicts. In our study, there was no difference in echocardiography measurements such as LVEDd, LVESd, mitral valve EF slope, mitral valve E/A ratios, and aortic and pulmonary valve VTI values between the groups. Our study showed that addiction is more of an electrical problem than a structural one, as evidenced by normal echocardiographic findings. Nevertheless, the noteworthy aspect of our study is the significant QTc prolongation in thinner addicts. Furthermore, our study also revealed an increase in P‐wave width in thinner users. The expansion of the P‐wave, which represents atrial depolarization (mean: 118.28, standard deviation: 10.89, Mann–Whitney *U*, *p* < 0.001), may serve as a predictive factor for the development of arrhythmias by impacting atrial electrical activity.

This study has some limitations. The participation motivation of thinner addicts in the study was less than ideal, and various challenges were encountered. There could be variations among observers and measurements. To investigate the reflection of electrical changes in the heart due to thinner addiction in detail, echocardiography can be extended to other parameters (such as tissue Doppler examinations, right ventricular functions, pulmonary artery pressures, etc.). As for ECG, QTc dispersion, corrected P‐wave dispersion, and signal‐averaged ECG testing can be conducted.

In conclusion, our study compared asymptomatic young adults with thinner addiction and the control group and revealed that individuals with thinner addiction exhibited increased uncorrected QT (*p* = 0.01), QTc (*p* = 0.003), and P‐wave width (*p* < 0.001). No significant differences were found in the echocardiographic measurements. As discussed, the occurrence of arrhythmic disturbances, sudden death syndrome, coronary vasospasm, syncopal episodes, and similar clinical conditions in those using thinner indicates that prolonged QTc and increased P‐wave width could be useful predictors. Although our study had a limited number of patients, it is the first to show the extension of the QTc interval in young adults dependent on thinner, even without symptoms. More research with larger patient groups using additional methods and examinations is necessary to make QTc and P‐wave width parameters standard practice for both diagnosis and treatment planning.

## Ethics Statement

Ethical approval for this study was granted by the Clinical Research Ethics Committee of Dr. Siyami Ersek Thoracic and Cardiovascular Surgery Education and Research Hospital.

## Conflicts of Interest

The authors declare no conflicts of interest.

## Data Availability

The data that support the findings of this study are available from the corresponding author upon reasonable request.
